# Attachment, Growth, and Detachment of Human Mesenchymal Stem Cells in a Chemically Defined Medium

**DOI:** 10.1155/2016/5246584

**Published:** 2016-02-23

**Authors:** Denise Salzig, Jasmin Leber, Katharina Merkewitz, Michaela C. Lange, Natascha Köster, Peter Czermak

**Affiliations:** ^1^Institute of Bioprocess Engineering and Pharmaceutical Technology, University of Applied Sciences Mittelhessen, 35390 Giessen, Germany; ^2^Faculty of Biology and Chemistry, Justus Liebig University, 35390 Giessen, Germany; ^3^Project Group Bioresources, Fraunhofer Institute for Molecular Biology and Applied Ecology (IME), 35394 Giessen, Germany; ^4^Department of Chemical Engineering, Kansas State University, Manhattan, KS 66506, USA

## Abstract

The manufacture of human mesenchymal stem cells (hMSCs) for clinical applications requires an appropriate growth surface and an optimized, preferably chemically defined medium (CDM) for expansion. We investigated a new protein/peptide-free CDM that supports the adhesion, growth, and detachment of an immortalized hMSC line (hMSC-TERT) as well as primary cells derived from bone marrow (bm-hMSCs) and adipose tissue (ad-hMSCs). We observed the rapid attachment and spreading of hMSC-TERT cells and ad-hMSCs in CDM concomitant with the expression of integrin and actin fibers. Cell spreading was promoted by coating the growth surface with collagen type IV and fibronectin. The growth of hMSC-TERT cells was similar in CDM and serum-containing medium whereas the lag phase of bm-hMSCs was prolonged in CDM. FGF-2 or surface coating with collagen type IV promoted the growth of bm-hMSCs, but laminin had no effect. All three cell types retained their trilineage differentiation capability in CDM and were detached by several enzymes (but not collagenase in the case of hMSC-TERT cells). The medium and coating did not affect detachment efficiency but influenced cell survival after detachment. CDM combined with cell-specific surface coatings and/or FGF-2 supplements is therefore as effective as serum-containing medium for the manufacture of different hMSC types.

## 1. Introduction

Human mesenchymal stem/stromal cells (hMSCs) are often used for cell therapy because they offer many advantageous characteristics [1]. Before therapeutic use, hMSCs must be expanded to produce the number of cells needed per patient and per dose (at least 1-2 × 10^6^ hMSCs per kg) [2]. The growth of hMSCs is anchorage-dependent, and the interactions among the growth surface, cells, and surrounding medium are therefore important for the manufacture of suitable numbers of healthy cells.

Cell adhesion is necessary for hMSC expansion and is driven by both nonspecific and specific interactions. Rounded cells in suspension initially attach to the surface due to complementary electrostatic/ionic forces and the growth surface then interacts with cell surface integrins, the principal receptors mediating cell-matrix adhesion [3]. Integrin activation results in the formation of heterodimers, which initiate signaling cascades that activate downstream genes and ultimately regulate cell morphology and behavior. The cell flattens and spreads due to the activation of protein kinase C (PKC) and the subsequent accumulation of focal adhesion kinase (FAK) and actin filaments at the leading edges of the cells. The completion of cell spreading and strong adhesion to the surface, which is required for proliferation, is characterized by the inactivation of PKC and the cross-linking of actin to defined intracellular stress fibers along with FAK located at the focal adhesion sites. The actin forms a stable cytoskeleton, which maintains the cell in its adherent spread state [4, 5].

Serum (usually bovine, sometimes human) is often included in hMSC expansion media to promote cell adhesion because it contains many attachment-promoting proteins (e.g., collagens, fibronectin, laminins, and vitronectin) as well as hormones and lipids that stimulate cell proliferation* in vitro* [6]. Serum causes problems when hMSC expansion must be carried out according to good manufacturing practice (GMP) because hMSCs in the clinic are considered advanced therapy medicinal products (ATMPs) by the European Medicines Agency (EMA) and US Food and Drug Administration (FDA). These agencies recommend the avoidance of any raw materials derived from mammals, including serum, to reduce the risk of contamination when using ATMPs [7].

The regulatory pressure to eliminate serum has resulted in several innovations [8]. In addition to serum-containing medium (SCM, 10–20% serum) and reduced serum medium (1–5% serum, fortified with insulin, transferrin, and other nutrients), several categories of serum-free medium have been developed including (a) serum-free medium, with additional mammalian hormones, growth factors, proteins, and polyamines; (b) protein-free medium, containing peptide fragments from the enzymatic or acid hydrolysis of proteins derived from animals or plants; (c) recombinant xeno-free medium, containing recombinant proteins/hormones/compounds and chemically defined lipids; and (d) chemically defined medium (CDM) which is a protein-free basal medium containing only low-molecular-weight additives, synthetic peptides or hormones, and a few recombinant or synthetic versions of proteins. Several in-house serum-free media have been developed for hMSC expansion, and these often contain additional factors such as bovine/human serum albumin, insulin, transferrin, hormones (e.g., progesterone, hydrocortisone, and estradiol), growth factors (e.g., bFGF, TGF-*β*, EGF, or PDGF), or heparin [9–18]. Commercial products are also available for this purpose, and although the full ingredient lists are not disclosed they also tend to include a selection of the components listed above. To our knowledge, however, the only protein/peptide-free CDM for hMSC expansion is the StemCell1 medium from Cell Culture Technologies, which completely lacks any proteinaceous components and each component is a defined concentration of a low-molecular-weight compound (between 50 and 250 Da, except one >1000 Da) that can be identified by its Chemical Abstracts Service registration number.

The absence of growth and attachment-promoting proteins in CDM may necessitate the use of protein coatings on the surface of tissue culture plasticware to encourage cell adhesion. Many serum proteins can be used as coatings, including native or denatured collagen, fibronectin, laminin, and vitronectin. Each protein is recognized by specific integrin heterodimers: native collagen by *α*_1_ *β*_1_, *α*_2_ *β*_1_, *α*_11_ *β*_1_, and *α*_Ib_ *β*_3_; denatured collagen by *α*_5_ *β*_1_, *α*_v_ *β*_3_, and *α*_IIb_ *β*_3_; fibronectin by *α*_2_ *β*_1_, *α*_3_ *β*_1_, *α*_4_ *β*_1_, *α*_4_ *β*_7_, *α*_5_ *β*_1_, *α*_8_ *β*_1_, *α*_v_ *β*_1_, *α*_v_ *β*_3_, *α*_v_ *β*_5_, *α*_v_ *β*_6_, *α*_v_ *β*_8_, and *α*_IIb_ *β*_3_; laminin by *α*_1_ *β*_1_, *α*_2_ *β*_1_, *α*_6_ *β*_1_, *α*_7_ *β*_1_, *α*_6_ *β*_4_, and *α*_v_ *β*_3_; and vitronectin by *α*_v_ *β*_1_, *α*_v_ *β*_3_, *α*_v_ *β*_5_, and *α*_IIb_ *β*_3_ [19]. The hMSCs isolated from bone marrow express the integrin subunits *α*_1_, *α*_2_, *α*_3_, *α*_5_, *α*_7_, *α*_8_, *α*_11_, *α*_v_, *β*_1_, *β*_3_, and *β*_5_ [20] and potentially also *α*_4_ and *α*_6_ [21]. Integrin expression in hMSCs differs by source, but hMSCs should bind via integrin receptors to each of the coatings listed above.

We investigated the attachment and spreading behavior of an immortalized hMSC cell line (hMSC-TERT) and two types of primary hMSCs derived from bone marrow (bm-hMSCs) and adipose tissue (ad-hMSCs) in the CDM StemCell1. We tested different protein coatings to determine which were able to promote the adhesion and growth of these three cell types in CDM. We also determined whether cells growing on the different coatings differ in terms of their detachment behavior and response to different detachment enzymes.

## 2. Materials and Methods

### 2.1. Cell Lines

We used primary hMSCs from bone marrow (bm-hMSCs, passages 3–10) kindly provided by M. Rook, Merck Millipore, Bedford, MA, USA, and from adipose tissue (ad-hMSCs, passages 3–10) kindly provided by F. Ehlicke, University of Würzburg, Würzburg, Germany. The immortalized cell line hMSC-TERT [22] (passages 74–80) was kindly provided by M. Kassem, University of Southern Denmark, Odense, Denmark.

### 2.2. Media

We used Eagle's minimal essential medium (EMEM) supplemented with 2 mM L-glutamine and 10% standardized fetal bovine serum (FBS, Article no. S0615) as our standard SCM. We used StemCell1 medium (Cell Culture Technologies, Gravesano, Switzerland) supplemented with 2 mM L-glutamine as our CDM. The media were further supplemented with 8 ng/mL recombinant human basic fibroblast growth factor (bFGF; Article no. W1370950050) when required. Unless otherwise specified, all components were purchased from Biochrom (Berlin, Germany).

### 2.3. Routine Cell Expansion and Adaption to CDM

Cryoconserved hMSC-TERT cells (10% DMSO, 90% FBS) and primary hMSCs were thawed and cultivated in tissue flasks (Sarstedt, Nümbrecht, Germany) containing EMEM with seeding densities between 5000 and 10,000 cells cm^−2^ at 37°C, in a 5% CO_2_ humidified atmosphere. Passaging was carried out at 80–90% confluence using 0.25 mg mL^−1^ trypsin-EDTA. CDM adaptation after the first passage was carried out using a mixture of 50% SCM and 50% CDM, and subsequently 100% CDM was used in Advanced TC*™* tissue flasks (Greiner Bio-One, Kremsmünster, Austria). All subsequent passaging was carried out using 25% conditioned medium from earlier cultures. The medium was replaced with 50% fresh medium every 3-4 days. Cells were passaged at least twice in CDM alone before starting the experiments.

### 2.4. Coating the Six-Well Plates

Collagen IV (human, C7521), fibronectin (bovine, F1141), laminin (murine, L2020), and vitronectin (human, SRP3186) were obtained from Sigma-Aldrich Laborchemikalien GmbH (Seelze, Germany). Each protein was applied to six-well plates overnight at 4°C to achieve a coating density of 5 *μ*mol cm^−2^. A set of plates was coated with 10% (v/v) FBS as a positive control.

### 2.5. Analysis of Cell Attachment and Spreading

The cells were suspended either in SCM or in CDM and plated with 7000 (hMSC-TERT), 8000 (bm-MSCs), or 3000 (ad-MSCs) cells cm^−2^ in coated or noncoated six-well plates. Attachment was analyzed for 5 h by counting the adherent and suspended cells every hour. Spreading was analyzed by counting the spread cells on microscopic images and defined as previously described [24].

### 2.6. Immunofluorescence Staining of the Cytoskeleton and Cell Surface Integrin *α*_*4*_

Cells were grown for 24 h either in SCM or in CDM on coated or noncoated six-well plates. We fixed the cells by removing the medium, gently washing with 2 mL PBS, and incubating with acetone (Carl Roth, Karlsruhe, Germany) for 10 min at −20°C. After two washes with PBS, the sample was incubated with 2 mL blocking solution (10 mg mL^−1^ BSA in PBS) for 30 min at room temperature. The sample was again washed twice and then incubated with a 1 : 80 dilution of Alexa Fluor® 555 Phalloidin (Life Technologies, Darmstadt, Germany, A340555) or a 1 : 200 dilution of DyLight 488 integrin *α*_4_ antibody MM0417-2L30 (R&D Systems GmbH, Wiesbaden, Germany, NBP2-11738G) in PBS for 2 h at room temperature in the dark. Finally, the nuclei were counterstained with DAPI (AppliChem, Darmstadt, Germany) and the sample was embedded in Mowiol (Carl Roth) according to the manufacturers' recommendations.

### 2.7. Analysis of Cell Growth

The cells were seeded in six-well plates (coated or noncoated) at a density of 7000-10,000 cells cm^−2^ and grown in 2 mL SCM or CDM for up to 8 d at 37°C in a 5% CO_2_ humidified atmosphere. Cells were counted under the microscope every day. The growth rate *μ* was determined during the exponential growth phase using the following equation:

(1) $$\begin{array}{c} \mu \left[\;\frac{1}{h}\;\right]=\frac{\ln\left(X_{2}\right)-\ln\left(X_{1}\right)}{t_{2}-t_{1}}. \end{array}$$

### 2.8. Differentiation of Expanded Cells

The cells were differentiated using StemMACS AdipoDiff, ChrondroDiff, or OsteoDiff media (Miltenyi Biotec, Bergisch Gladbach, Germany) according to the manufacturer's recommendations. After differentiation, the cells were fixed with 4% paraformaldehyde (Carl Roth) for 30 min at room temperature. Adipogenic differentiation was confirmed by nil red staining of the fat droplets as previously described [25]. Finally, the sample was embedded with Mowiol (Carl Roth) according to the manufacturer's recommendations. Chondrogenic differentiation was confirmed by the immunofluorescence staining of collagen type II as described [26]. Osteogenic differentiation was confirmed using the OsteoImage Mineralization Assay (Lonza, Basel, Switzerland) according to the manufacturer's recommendations.

### 2.9. Analysis of Cell Detachment

The cells were suspended in SCM or CDM, seeded at a density of 50,000 cells cm^−2^ in coated or noncoated six-well plates and incubated at 37°C in a 5% CO_2_ humidified atmosphere until attachment was observed. Each well was washed twice with 1 mL PBS to remove the medium. The cells were incubated with 0.5 mL detachment enzyme solution (supplemented with 0.02% EDTA if necessary) as shown in Table 1. The detachment reaction was stopped by adding 1.5 mL SCM, the solution was centrifuged (500 × g, 5 min, room temperature), and the remaining cell pellet was resuspended in 0.5 mL SCM. Cell number and viability were determined by trypan blue staining.

## 3. Results

### 3.1. Attachment and Spreading of hMSC-TERT Cells and Primary hMSCs

**Figure 1:**
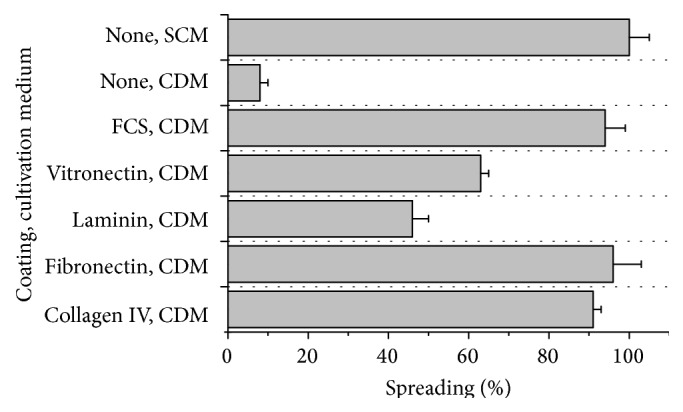
Spreading of hMSC-TERT cells on different surface coatings. The hMSC-TERT cells were grown on different coatings either in serum-containing medium (SCM) or in chemically defined medium (CDM). The cells were analyzed by microscopy and those showing at least three lamellipodia were defined as spread. Each measurement was taken in triplicate (*n* = 3).

**Figure 2:**
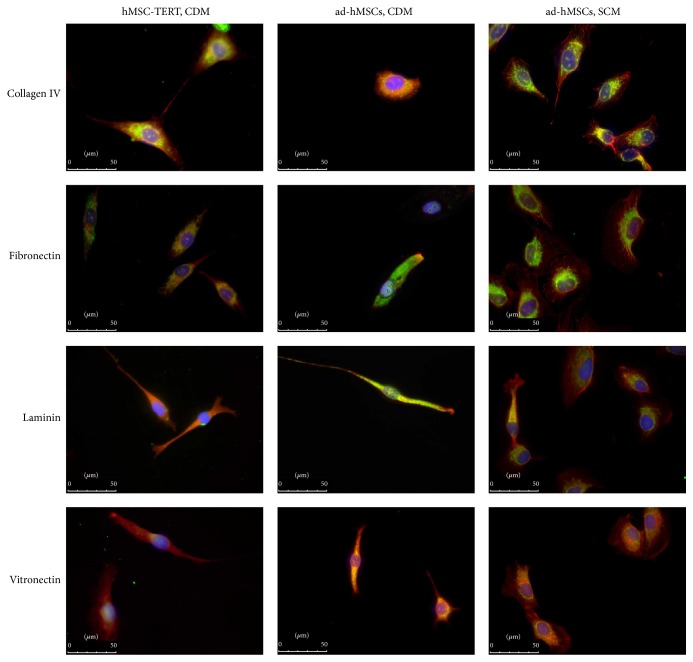
Immunofluorescence staining of the cytoskeleton and the cell surface integrin *α*_4_ in hMSC-TERT cells and primary adipose-derived hMSCs (ad-hMSCs). The cells were grown on different coatings either in serum-containing medium (SCM) or in chemically defined medium (CDM). After fixation, immunofluorescence staining was carried out showing F-actin in red, integrin *α*_4_ in green, and nuclei in blue.

Efficient cell attachment and spreading on the growth surface are necessary to expand anchorage-dependent hMSCs. In CDM, the hMSC-TERT cells completely attached within 4 h regardless of the presence/absence or type of surface coating. We observed minor surface-dependent differences in the attachment rate; for example, the cells attached more slowly on the collagen IV coating. Nevertheless, there was little difference in the attachment behavior of hMSC-TERT cells growing in SCM and CDM. In contrast, the spreading of the cells in CDM was positively influenced by the protein coatings. In the absence of coating, only 8% of the attached cells spread after 5 h, whereas cells seeded on collagen type IV and fibronectin spread at similar rates to those seeded on FBS or in SCM (Figure 1). The immunofluorescence staining of the cytoskeleton and cell surface integrins revealed that hMSC-TERT cells growing in CDM on surfaces coated with collagen type IV or fibronectin contained better-organized F-actin fibers than cells growing on other surfaces and also expressed integrin *α*_4_ at a higher level (Figure 2).

**Figure 3:**
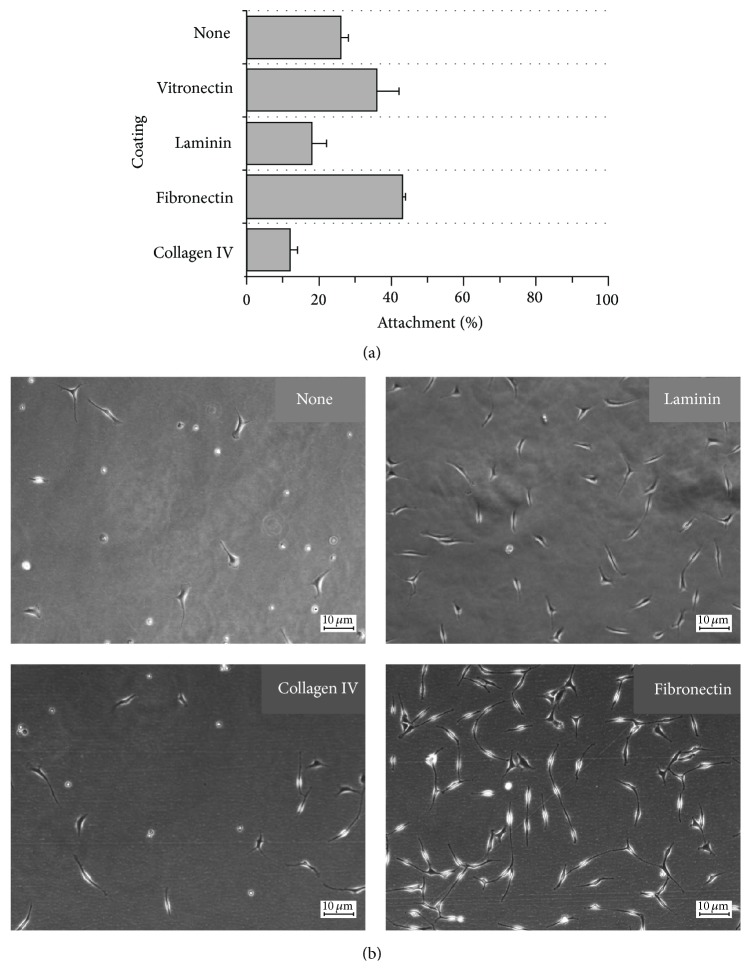
Attachment and spreading of primary bone marrow-derived hMSCs (bm-hMSCs) in chemically defined medium (CDM). (a) Attachment was measured by counting the adherent and suspended cells. (b) The cells were analyzed by microscopy and those showing at least three lamellipodia were defined as spread. Each measurement was taken in triplicate (*n* = 3).

The bm-hMSCs attached poorly in CDM (Figure 3(a)) but even in SCM complete attachment took up to 24 h. Only a few bm-hMSCs had attached after 5 h in CDM and no spreading was observed within this time period. The attached bm-hMSCs were thin and elongated on each of the coatings and no lamellipodia were observed. Our results showed that no coating was preferable for the attachment or spreading of these cells in CDM, but without coating the attached cells tended to detach again and become rounded (Figure 3(b)).

In contrast, the ad-hMSCs attached rapidly in CDM and more than 90% of the cells had attached within 2 h, regardless of the presence/absence or type of coating. Within 4 h, 59% of the cells had spread on the collagen type IV surface whereas almost 100% of cells had spread on the fibronectin, laminin, and vitronectin surfaces after the same amount of time. The immunofluorescence staining showed that all the expanded ad-hMSCs in CDM expressed integrin *α*_4_, but the F-actin fibers were not as well organized and distinct as those observed in the cells cultivated in SCM (Figure 2).

### 3.2. Growth of hMSC-TERT Cells and Primary hMSCs

**Figure 4:**
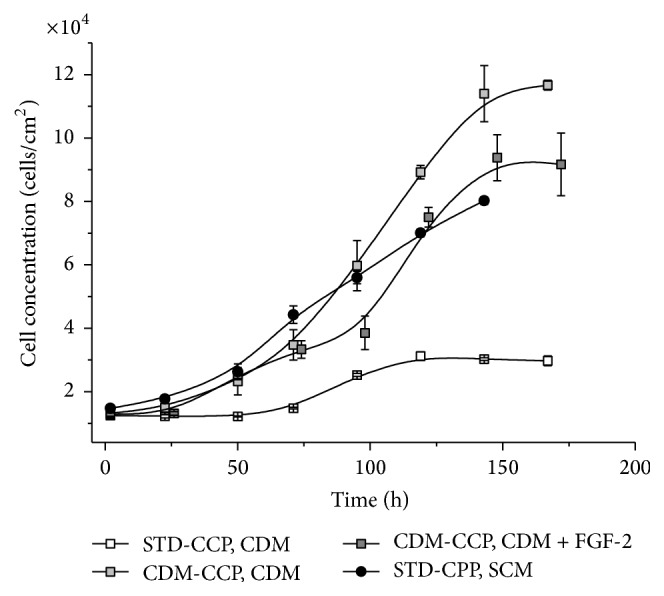
Growth of hMSC-TERT cells in chemically defined medium (CDM). The cells were grown on standard cell culture plastic (STD-CCP) or CDM-optimized CCP (CDM-CCP) in either CDM (with or without FGF-2) or serum-containing medium (SCM). Cell growth was analyzed by the counting and the measurement of glucose consumption. Each measurement was taken in triplicate (*n* = 3).

A comprehensive investigation of hMSC-TERT growth behavior in CDM initially showed that the choice of cell culture plastic (CCP) had an enormous influence (Figure 4). In standard CCP, the hMSC-TERT cells had a prolonged lag phase and a slower growth rate (*μ*_STD-CCP_ = 0.013 h^−1^) compared to cells growing in SCM (*μ*_SCM_ = 0.020 h^−1^). In addition, the maximum density of cells growing on standard CPP was 2.6-fold lower in CDM compared to SCM. On CPP specially designed for compatibility with CDM cultivation (CDM-CPP), the growth rate of the cells was similar in CDM and SCM (*μ*_CDM-CCP_ = 0.019 h^−1^). Supplementing the CDM with FGF-2 or coating the CPP with the proteins listed above did not improve the growth rate any further.

**Figure 5:**
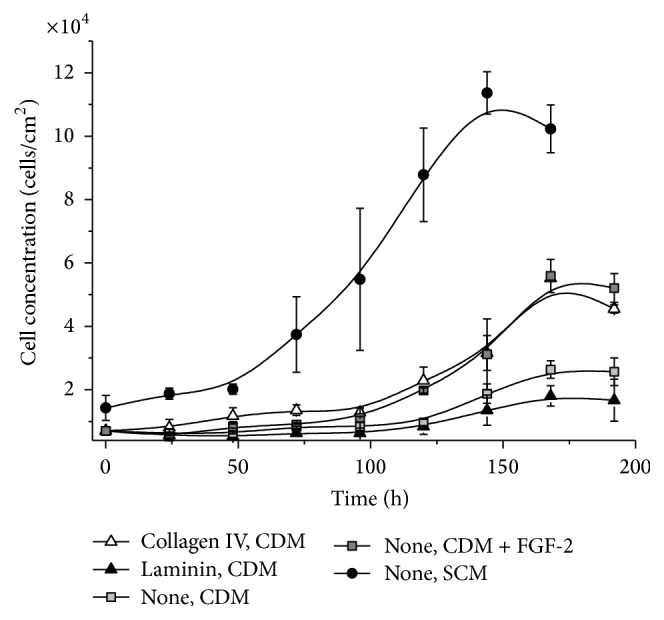
Growth of primary bone marrow-derived hMSCs (bm-hMSCs) in chemically defined medium (CDM). The cells were grown on coated or noncoated CDM-optimized cell culture plastic either in CDM (with or without FGF-2) or in serum-containing medium (SCM). Cell growth was analyzed by the counting and the measurement of glucose consumption. Each measurement was taken in triplicate (*n* = 3).

The growth of bm-hMSCs in CDM was only tested using CDM-CPP. We found that cell growth was much slower in the absence of coating (*μ*_CDM-CCP_ 0.016 h^−1^) when compared to the growth rate in SCM (*μ*_SCM_ 0.020 h^−1^), and that the cell number at the end of the cultivation was four times lower in CDM compared to SCM (Figure 5). For both primary hMSCs, supplementing the CDM with FGF-2 significantly improved the cell growth rate (*μ*_FGF2_ = 0.019 h^−1^), but the cell number at the end of the cultivation in CDM was only half that achieved using SCM. This probably reflects the duration of the lag phase, which is 48 h longer in CDM compared to SCM. The nature of coating also affected the growth rate of bm-hMSCs in CDM; for example, laminin did not promote cell growth any better than uncoated plates, whereas collagen type IV improved the bm-hMSC growth rate to the same extent as supplementing the medium with FGF-2.

### 3.3. Differentiation Potential of hMSC-TERT Cells and Primary hMSCs in CDM

**Figure 6:**
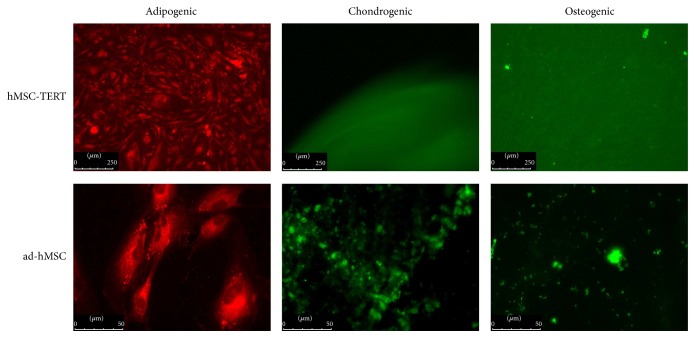
Differentiation capacities of hMSC-TERT cells and primary adipose-derived hMSCs (ad-hMSCs). The cells were induced to undergo adipogenic, chondrogenic, or osteogenic differentiation in commercial media after expansion in CDM. Differentiation was confirmed by nil red staining (red, adipogenic), collagen type II immunostaining (green, chondrogenic), or hydroxyapatite staining (green, osteogenic). The primary bone marrow-derived hMSCs (bm-hMSCs) behaved in a similar manner (data not shown).

Trilineage differentiation potential is a minimal criterion for the therapeutic use of hMSCs, so we investigated whether the expansion of cells in CDM had any influence on this property. We found that hMSC-TERTs, bm-hMSCs, and ad-hMSCs each retained their ability to differentiate into adipocytes, chondrocytes, and osteoblasts, as determined by immunofluorescence staining (Figure 6).

### 3.4. Detachment of hMSC-TERT Cells and Primary hMSCs

**Figure 7:**
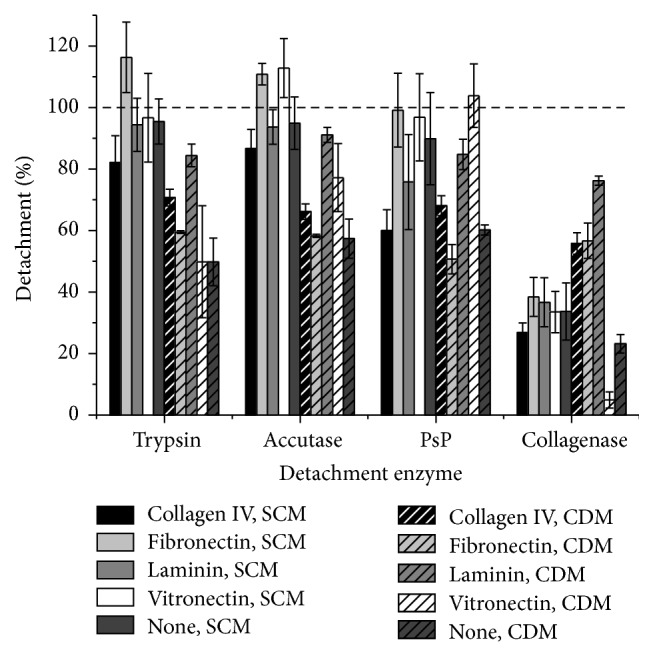
Detachment of hMSC-TERT cells using different enzymes. The cells were grown to confluency in coated or noncoated wells and were detached enzymatically. Cell detachment was analyzed by counting cells in suspension. Each experiment was carried out in triplicate (*n* = 3).

**Figure 8:**
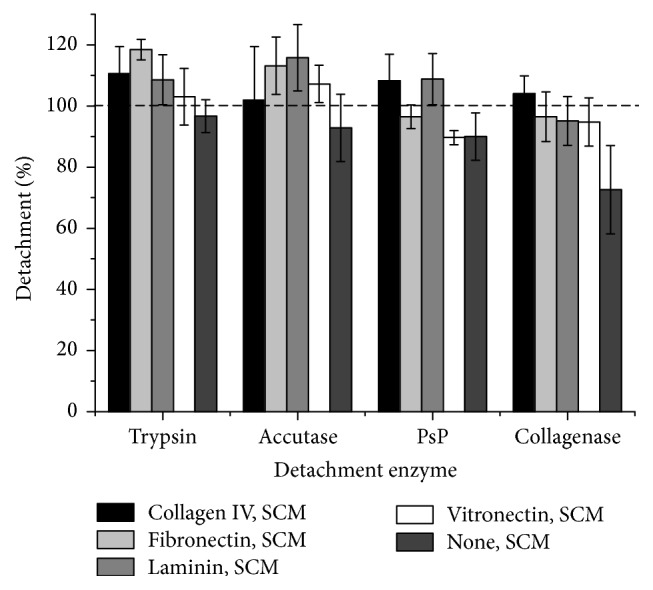
Detachment of primary bone marrow-derived hMSCs (bm-hMSCs) using different enzymes. The cells were grown to confluency in coated or noncoated wells and were detached enzymatically. Cell detachment was analyzed by counting cells in suspension. Each experiment was carried out in triplicate (*n* = 3).

Detachment is also necessary for hMSC expansion and this process should be efficient without causing cell damage. Compared to hMSC-TERT cells grown in SCM, we found that the same cells growing in CDM were more difficult to detach with trypsin, Accutase, and PsP and that collagenase was completely ineffective even if the surface of the flasks was coated with collagen (Figure 7). For hMSC-TERT cells grown in SCM, the coating had no influence on detachment with trypsin or Accutase because both enzymes achieved almost 100% detachment. For hMSC-TERT cells grown in CDM, trypsin and Accutase detached most cells from laminin-coated surfaces. PsP was unable to detach hMSC-TERT cells from surfaces coated with collagen type IV in either SCM or CDM. This enzyme efficiently removed cells from all other surfaces in both media, but the efficiency of cell detachment from fibronectin was 50% lower in CDM compared to SCM. Although CDM generally had little impact on detachment efficiency, it did affect the viability of the detached cells. For hMSC-TERT cells grown in CDM without a surface coating, the viability fell substantially after detachment with trypsin (80.7 ± 3.3%), Accutase (87.2 ± 2.0%), and collagenase (83.8 ± 9.2%) but remained high after detachment with PsP (97.4 ± 0.1%). In contrast, the cells grown without a surface coating in SCM only lost viability following detachment with collagenase (88.6 ± 1.9%). The bm-hMSCs could be detached efficiently from each coating with any of the enzymes, including collagenase. We observed no differences among the four coatings, but detachment was slightly less efficient in the absence of a coating. All detached cells remained highly viable after detachment (Figure 8).

## 4. Discussion

### 4.1. Interaction between Cells and the Growth Surface

The nature of the growth surface can have a profound effect on the behavior of cultured cells, and we found that this was also the case for three different types of hMSCs. Switching from SCM to CDM affected the growth of hMSCs on standard tissue culture plastic, but a specially modified surface designed for compatibility with CDM improved the growth of hMSC-TERT cells to the same extent as SCM, and coating this surface with extracellular matrix (ECM) proteins or adding FGF-2 to the medium did not improve growth any further. The modified plastic surface is prepared by incubating it with plasma, which provides more oxygen groups to increase wettability, protein interactions, and thereby cell proliferation [27]. The impact of further coating with ECM proteins differed according to the cell type. Fibronectin promoted the adhesion of hMSC-TERT cells and ad-hMSCs, which is not surprising because hMSCs express more fibronectin receptors than receptors for each of the three other coatings [20, 28]. Integrin *α*_4_ is a major fibronectin receptor [19] and we were able to detect this protein on the surface of these hMSCs grown on fibronectin in SCM and CDM. Interestingly, integrin *α*_4_ was also present on hMSC-TERT cells growing in CDM on collagen type IV although integrins *α*_1_, *α*_2_, *α*_10_, and *α*_11_ combine with integrin *β*_1_ to bind collagen IV and integrin *α*_4_ is not involved. Fibronectin and collagen IV are interconnected, and collagen type IV educates other ECM components and promotes the survival of fibroblasts and tumor cells independent of its integrin and specificity as a way to circumvent apoptosis [29, 30]. We observed this growth-promoting effect for bm-hMSCs growing in CDM on a collagen type IV coating. The coating had the same positive impact as the growth factor FGF-2, which is known to enhance the mitotic potential of hMSCs and increase their growth rate and potential for self-renewal [31, 32].

### 4.2. The Behavior of the Three Types of hMSC in CDM

The attachment and spreading kinetics of the three types of hMSC in CDM were strongly dependent on the cell type. Whereas the hMSC-TERT cells and ad-hMSCs attached and spread rapidly, the adhesion of the bm-hMSC was slow and inefficient. The latter were also slow to attach in SCM, which suggests the effect is cell- or donor-dependent rather than indicative of missing attachment-promoting proteins in the CDM. Human MSCs from different sources differ in their integrin profiles [33]; for example, hMSC-TERT cells express integrins *α*_2_, *α*_4_, *α*_5_, *α*_6_, *α*_11_, *α*_v_, *β*_1_, and *β*_5_ [28], whereas primary hMSCs express integrins *α*_1_, *α*_2_, *α*_3_, *α*_6_, *α*_7_, *α*_9_, *α*_11_, and *β*_1_ [34], and this is likely to affect their adhesion behavior. Importantly, cell vigor also depends on the age and health of the donor. The bm-hMSCs were derived from an older donor than the ad-hMSCs, which could explain their slow attachment and proliferation compared to the ad-hMSCs and the immortalized hMSC-TERT cells. To exclude donor-dependent effects and confirm that the observed behavior in CDM is genuinely cell-dependent, it will be necessary to repeat the experiments using bm-hMSCs and ad-hMSCs from at least five donors.

Strong adhesion is required for cell growth, so the inefficient adhesion of the bm-hMSCs in CDM may explain the long lag phase compared to the same cells grown in SCM. The growth rate was improved by supplementing the medium with FGF-2 or coating the surface with collagen type IV, but the lag phase could not be shortened. To confirm this hypothesis, the adhesion strength of the cells should be analyzed in future experiments, for example, by atomic force microscopy [35].

The detachment behavior of the three types of hMSC was also distinct and depended on the medium and the surface coating. For cells grown in CDM, surface coating did not improve the efficiency of detachment, but it did increase cell viability after cell detachment indicating a protective effect. Interestingly, the hMSC-TERT cells could not be detached with collagenase, even if the cells were grown on a collagen-coated surface, despite the fact that collagenase is often used to isolate hMSCs from tissues [36]. In previous studies, we showed that collagenase is not suitable for the detachment of hMSC-TERT cells from glass surfaces because it primarily cleaves cell-cell linkages and not cell surface linkages [37, 38]. In contrast to hMSC-TERT cells, the primary hMSCs could easily be detached with collagenase and the other enzymes.

### 4.3. Influence of Surface Coating and the Medium on Stem Cell Potency

All three hMSC types retained their stem cell phenotype and capacity for multilineage differentiation when expanded in CDM, showing that CDM is suitable for robust hMSC expansion and does not contain soluble factors that promote unwanted differentiation [39].

We did not determine whether the coating influences the potency of hMSCs, but this must be considered because certain ECM components can induce differentiation. For example, fibronectin promotes cell spreading and proliferation while inhibiting adipogenic differentiation, but it plays a pivotal role during osteogenic differentiation. Furthermore, vitronectin and collagen type I can promote osteogenic differentiation in hMSCs, whereas laminin can stimulate the proliferation of hMSCs (although we could not confirm this in our experiments) but suppresses chondrogenesis [40]. This shows that multiple ECM components can provide a suitable attachment and growth surface for hMSCs, but these must be chosen carefully to avoid unwanted differentiation during cell expansion.

## 5. Conclusions

The manufacture of hMSCs for clinical applications requires an appropriate choice of growth surface and expansion medium. We have demonstrated that it is possible to expand different primary hMSCs and an immortalized hMSC line in protein/peptide-free CDM, which means that fewer supplements are required than anticipated and that the cells can survive in a basic medium. The cultivation of cells for a few days or for one or two passages is not enough to declare a robust serum-free medium, because stem cells can proliferate in basal medium [41]. Therefore, it will be necessary to expand the hMSCs for longer periods to determine whether the CDM is suitable for manufacturing. Nevertheless, the behavior of each of the three cell types in CDM was distinct. For example, the fibronectin coating was only advantageous for ad-hMSC and hMSC-TERT attachment but did not affect bm-hMSCs. Furthermore, FGF-2 and collagen IV promoted the growth of bm-hMSCs but not hMSC-TERT cells. It is not yet possible to recommend a generally advantageous coating or supplement for the expansion of each MSC type in CDM because donor-dependent effects could not be excluded. Therefore, more extensive studies with hMSCs from other sources (e.g., umbilical cord) and with more donors per cell type are necessary to determine whether general principles can be drawn from these data. Efficient hMSC manufacturing requires a detailed understanding of the interactions among the cells, the growth surface, and the cultivation medium.

## Figures and Tables

**Table 1:** Properties of the enzymes used for cell detachment.

Detachment enzyme	Manufacturer	Incubation time (min)
Accutase	Sigma-Aldrich	10
Collagenase	PAA Laboratories GmbH	60
Prolyl-specific peptidase (PsP)	[23]	40
Trypsin	PAA	10
